# Prenatal Supplementation in Beef Cattle and Its Effects on Plasma Metabolome of Dams and Calves

**DOI:** 10.3390/metabo12040347

**Published:** 2022-04-13

**Authors:** Fernando José Schalch Junior, Guilherme Henrique Gebim Polizel, Fernando Augusto Correia Queiroz Cançado, Arícia Christofaro Fernandes, Isabela Mortari, Pedro Ratto Lisboa Pires, Heidge Fukumasu, Miguel Henrique de Almeida Santana, Arlindo Saran Netto

**Affiliations:** 1 Department of Animal Science, Faculty of Animal Science and Food Engineering, University of Sao Paulo, Av. Duque de Caxias Norte, 225, Pirassununga 13635-900, SP, Brazil; fernando@minerthal.com.br (F.J.S.J.); guilhermepolizel875@gmail.com (G.H.G.P.); aricia.fernandes@usp.br (A.C.F.); isa_mortari@hotmail.com (I.M.); saranetto@usp.br (A.S.N.); 2 Department of Basic Sciences, Faculty of Animal Science and Food Engineering, University of Sao Paulo, Av. Duque de Caxias Norte, 225, Pirassununga 13635-900, SP, Brazil; facqc@usp.br; 3 Department of Veterinary Medicine, College of Animal Science and Food Engineering, University of Sao Paulo, Av. Duque de Caxias Norte, 225, Pirassununga 13635-900, SP, Brazil; pedroratto@gmail.com (P.R.L.P.); fukumasu@usp.br (H.F.)

**Keywords:** beef cattle, fetal programming, mass spectrometry, maternal nutrition, metabolites

## Abstract

This study investigated the effect of different prenatal nutrition on the plasma metabolome of Nellore dams and their offspring. For that purpose, three nutritional treatments were used in 126 cows during pregnancy: NP—(control) only mineral supplementation; PP—protein-energy supplementation in the final third; and FP—protein-energy supplementation during the entire pregnancy. Targeted metabolomics were analyzed in plasma at the beginning of pregnancy and in pre-delivery of cows (*n* = 27) as well as in calves (*n* = 27, 30 ± 9.6 days of age). Data were analyzed by the analysis of variance, partial least squares discriminant analysis, and the principal component analysis (PCA). The PCA showed a clear clustering in the periods investigated only in cows (early gestation and pre-delivery). We found significant metabolites in both supervised analyses (*p* < 0.05 and VIP score > 1) for cows (Taurine, Glutamic acid, Histidine, and PC aa C42:2) and for calves (Carnosine, Alanine, and PC aa C26:0). The enrichment analysis revealed biological processes (*p* < 0.1) common among cows and calves (histidine metabolism and beta-alanine metabolism), which may be indicative of transgenerational epigenetic changes. In general, fetal programming affected mainly the metabolism of amino acids.

## 1. Introduction

Maternal nutrition during pregnancy has received great prominence in livestock studies because of its effects on the production traits of dams and long-term consequences on the offspring [1,2,3,4]. Undernutrition or overnutrition during the gestational period can cause physiological and metabolic changes to the fetus that can last throughout its productive life [5,6]. Several studies have reported the phenotypic effects on offspring due to different prenatal stimuli in beef cattle from extensive production systems [7,8,9,10,11,12]. Many of these phenotypic consequences are related to carcass characteristics, meat quality, body composition, weight gain, body weight, and reproductive performance. However, the molecular mechanisms involved need further elucidation.

With recent advances in “omics” technologies, holistic molecular approaches, such as metabolomics, are more accessible and can contribute to a better understanding of the molecular mechanisms involved with the phenotype of interest. Metabolome is defined as a complete collection of metabolites in a specific organ, tissue, cell, organelle, or biofluid [13]. Metabolites can be sensitive indicators of changes in the genome, transcriptome, and/or proteome, as they are the product of complex interactions between different molecular levels [14]. Thus, the metabolomics approach shows great potential to analyze the metabolic and physiological effects related to a phenotype [15].

Some studies have used a metabolomics perspective to assess environmental effects and phenotypes on livestock [16,17,18,19]. However, few studies have investigated metabolomics approaches in experiments that evaluate maternal nutrition and its effects on offspring in beef cattle [20,21].

We hypothesize that different protein-energy supplementation strategies could change the metabolic profile of cows during pregnancy and thus of their progenies. Here, we evaluated whether the different prenatal nutritional conditions affect the metabolic profile (amino acid, biogenic amines, hexose, acylcarnitines, lysophosphatidylcholines, phosphatidylcholines, and sphingolipids) of the blood plasma of cows and their respective offspring using the targeted metabolomics approach.

## 2. Results

### 2.1. Univariate Analysis of Metabolome

Initially (early gestation), cows had five differentially expressed metabolites between prenatal treatments (PC aa C30:0; Histidine; PC ae C30:2; PC ae C30:0; PC ae C30:1; Table 1). In the evaluation of pre-delivery metabolomics, the cows presented 16 differentially expressed metabolites between the groups (Symmetric dimethylarginine (SDMA); PC aa C34:4; PC aa C38:3; PC aa C40:3; Taurine; Glutamic acid; PC ae C38:1; PC ae C34:3; PC aa C40:4; PC aa C42:4; PC aa C42:2; PC ae C42:4; PC aa C42:6; Histidine; Carnosine; PC aa C36:2; Table 2). Regarding the metabolomes of calves, six differentially expressed metabolites between the treatments (PC aa C42:6; PC ae C38:4; Carnosine; Alanine; PC aa C26:0; PC ae C40:4; Table 3). The tables with all metabolites (significant and not significant) and respective *p* values can be found in Supplementary Material (Tables S1–S3).

### 2.2. Principal Component Analysis (PCA)

**Figure 1:**
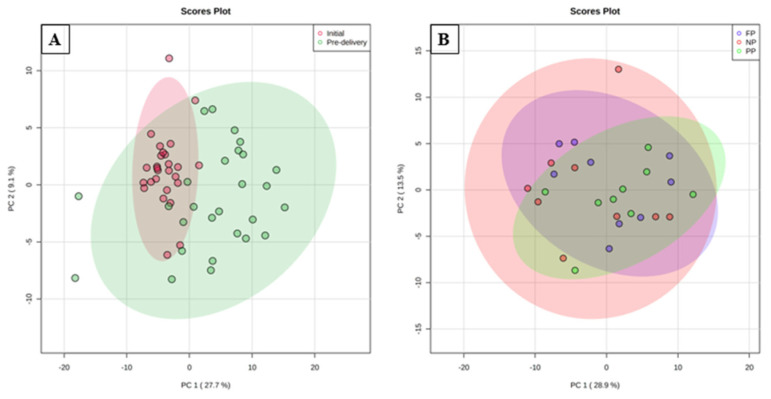
Principal component analysis (PCA) scores plot of metabolome distribution of plasma of dams between the times ((**A**) initial and pre-delivery) and between the nutritional treatments in pre-delivery period ((**B**) NP, PP and FP).

The PCA results of the different periods analyzed for dams (Figure 1A) showed that data distribution presented a clustering for the initial period and another for the pre-delivery period. Thus, the data from the initial period were more homogeneous, indicating more similar metabolic levels than in the pre-delivery period (after receiving nutritional treatments). The two principal components together explained 36.8% of the total variance (PC1 = 27.7%; PC2 = 9.1%).

The PCA of treatments in pre-delivery of dams (Figure 1B) showed a data distribution with a partial overlap between all groups. This may indicate that the metabolite profile did not present a large number of differentially expressed variables between the treatments. The two principal components together explained 42.4% of the total variance (PC1 = 28.9%; PC2 = 13.5%).

**Figure 2:**
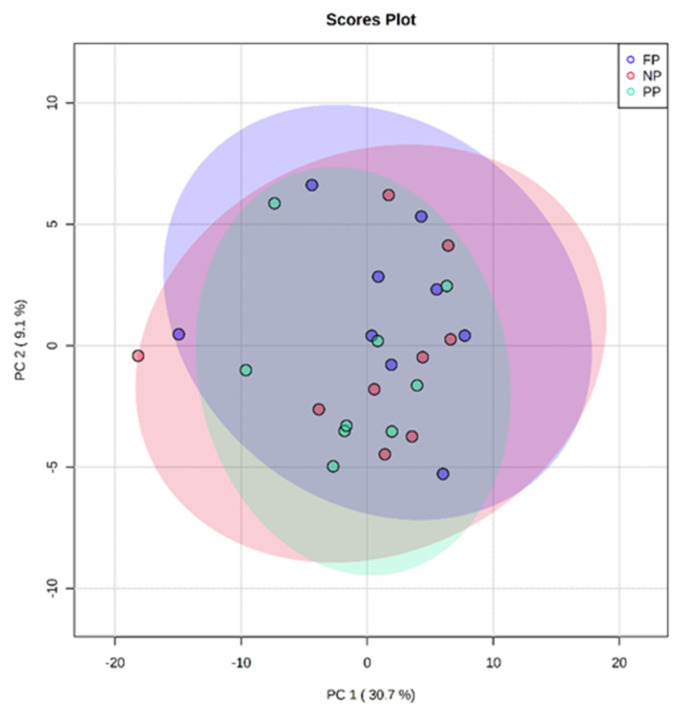
Principal component analysis (PCA) scores plot of metabolome distribution of plasma of offspring between the prenatal nutritional treatments at 30 days of age (NP, PP, and FP).

The PCA of treatments in calves at 30 days of age (Figure 2) showed a data distribution with an overlap between all groups; however, it was not possible to observe a clustering between the treatments. This may indicate that the metabolite profile presented only a few or no differentially expressed variables between the treatments. The two principal components together explained 39.8% of the total variance (PC1 = 30.7%; PC2 = 9.1%).

### 2.3. Partial Least Squares Discriminant Analysis (PLS-DA)

**Figure 3:**
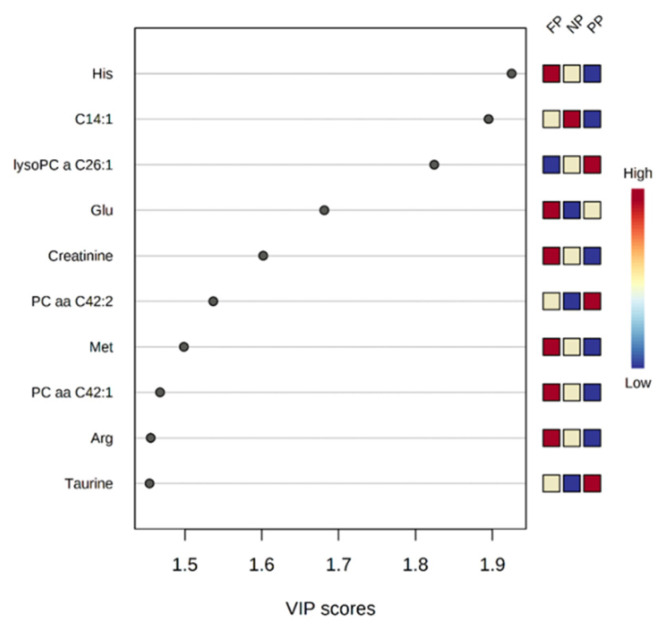
Top 10 metabolites (VIP scores) of dams in the pre-delivery stage associated to the different prenatal treatments (NP, PP and FP).

Based on variable importance in the projection (VIP) scores, compounds that contributed to a higher percentage of the residuals explained in the PLS-DA plot between the prenatal nutritional treatments in dams (pre-delivery; Figure 3) were: His (Histidine; VIP = 1.925), C14:1 (VIP = 1.895), lysoPC a C26:1 (VIP = 1.824), Glu (Glutamic acid; VIP = 1.681), Creatinine (VIP = 1.601), PC aa C42:2 (VIP = 1.536), Met (Methionine; VIP = 1.498), PC aa C42:1 (VIP = 1.467), Arg (Arginine; VIP = 1.455), and Taurine (VIP = 1.453).

**Figure 4:**
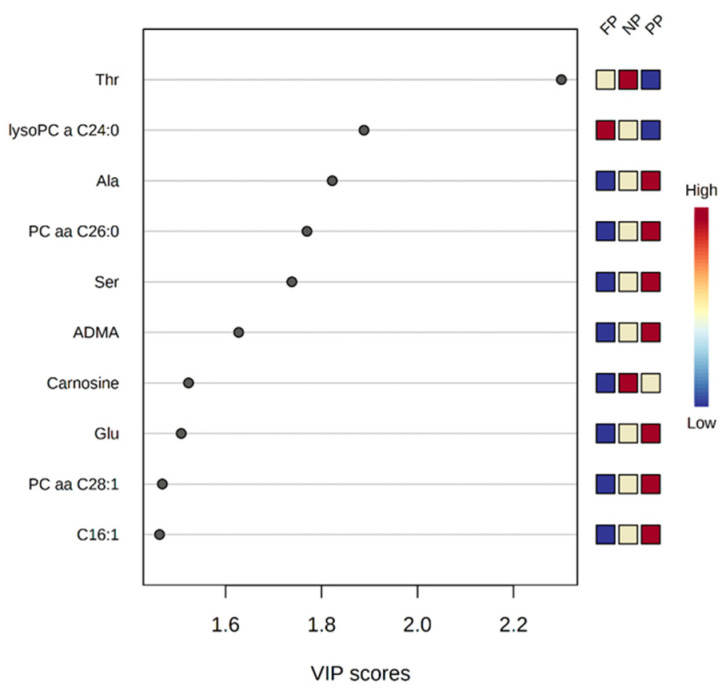
Top 10 metabolites (VIP scores) of calves at 30 days of age associated to the different prenatal treatments (NP, PP, and FP).

The top 10 metabolites, based on VIP scores, of calves at 30 days of age submitted to different prenatal nutritional treatments (Figure 4) were: Thr (Threonine; VIP = 2.299), lysoPC a C24:0 (VIP = 1.888), Ala (Alanine; VIP = 1.822), PC aa C26:0 (VIP = 1.769), Ser (Serine; VIP = 1.738), ADMA (Asymmetric dimethylarginine; VIP = 1.627), Carnosine (VIP = 1.522), Glu (VIP = 1.507), PC aa C28:1 (VIP = 1.468), and C16:1 (VIP = 1.462).

### 2.4. Enrichment Analysis

**Figure 5:**
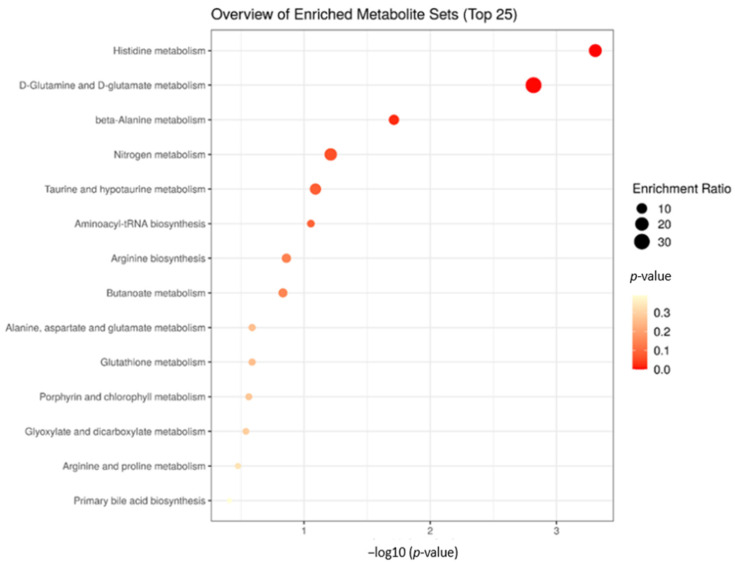
Biological processes involved with significant plasma metabolites of the dams in the pre-delivery stage after receiving the three nutritional treatments (NP, PP, and FP).

The enrichment analysis of dams in the pre-delivery period showed six significant biological processes related to differentially expressed metabolites between the prenatal treatments (Figure 5). The significant metabolic processes were: Histidine metabolism (*p* = 4.92 × 10^−4^), D-Glutamine and D-glutamate metabolism (*p* = 1.52 × 10^−3^, beta-Alanine metabolism (*p* = 0.02), Nitrogen metabolism (*p* = 0.06), Taurine and hypotaurine metabolism (*p* = 0.08), and Aminoacyl-tRNA biosynthesis (*p* = 0.08).

**Figure 6:**
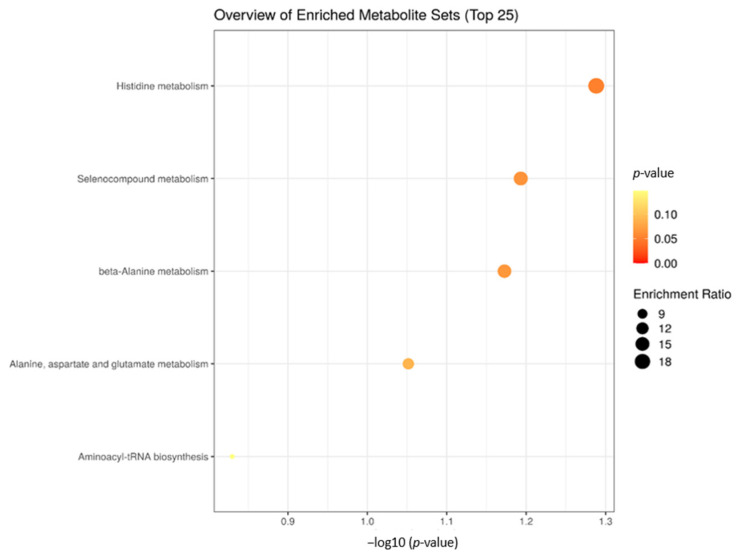
Biological processes involved with significant plasma metabolites of calves at 30 days of age submitted to the three prenatal nutritional treatments (NP, PP and FP).

The enrichment analysis of calves with an average age of 30 days showed four significant biological processes related to differentially expressed metabolites between the prenatal treatments (Figure 6). The significant metabolic processes were: Histidine metabolism (*p* = 0.05), Selenocompound metabolism (*p* = 0.06), beta-Alanine metabolism (*p* = 0.06), Alanine, aspartate, and glutamate metabolism (*p* = 0.09).

## 3. Discussion

To the best of our knowledge and based on a literature search, this is the first study that assessed the impact of three prenatal supplementation approaches on the plasma metabolome of beef cattle dams and their progenies.

The results showed that the initial period of dams had five differentially expressed metabolites between the groups. During this period, the animals showed the same initial phenotypic levels (see Table 6 in the methodology section) and clustering variables as the pre-delivery period in PCA (Figure 1A). This demonstrated that the different maternal nutrition approaches the affected plasma metabolome of the animals in the pre-delivery stage.

We selected the significant metabolites for discussion found in both supervised analyses (ANOVA and PLS-DA) and related to significant biological processes. Thus, as they were significant in both analyses, these metabolites were considered the main plasma metabolites involved in the different prenatal nutrition strategies in Nellore cattle. In pre-delivery of dams, the following metabolites were selected: Taurine, Glutamic acid, Histidine, and PC aa C42:2. In calves, the following were selected: Carnosine, Alanine, and PC aa C26:0.

Taurine is a sulfur-containing amino acid (non-essential amino acid) that can be found in the plasma of mammals after birth. This amino acid is synthesized in the liver and in the white adipose tissue. Low Taurine levels can be associated with alterations in glucose metabolism, insulin sensitivity, as well as the function and number of β-cells [22]. In a previous study, no difference was found in blood levels of Taurine between groups of cows (pre-delivery) with higher and lower body condition scores (BCS) [23]. In our study, Taurine levels showed differences between the NP and PP groups, which did not show differences in BCS in the pre-delivery period, but these groups received different protein and energy inputs. Protein-energy supplementation only in the final third of pregnancy (PP) showed higher Taurine levels in pre-delivery compared to dams that did not receive this nutritional stimulus in any gestational period (NP). Low levels of maternal Taurine may reduce the antioxidant activity, impairing progeny growth and perinatal development of the central nervous system and of the endocrine pancreas [24,25].

Glutamic acid is a functional amino acid that plays several roles in the intestinal tract [26], such as immune responses and function barriers [27,28,29]. It also plays specific roles as a source of energy for the intestinal mucosa [30], as a mediator of cell signaling [31], a regulator of oxidative processes [32], and a substrate for several metabolic pathways [33]. In another study, the glutamate metabolic pathway was one of the biological processes found among cows with divergent residual feed intake (low RFI and high RFI), demonstrating an association with feed efficiency in cows [34]. In our study, one of the significant metabolic pathways found between different prenatal treatments was also related to glutamic acid and glutamate (D-Glutamine and D-glutamate metabolism), showing that different supplementation approaches in cows may also affect this metabolic pathway.

Histidine is an essential amino acid in mammals and must thus be obtained through the diet [35]. Histidine deficiency can reduce the body weight (BW) of animals [36]. Specifically, in cattle, histidine deficiency is the first limiter for the growth of all amino acids in the duodenum [37]. The higher histidine levels also affect the cow milk yield, lactose yield, protein milk yield, fat milk proportion, dry matter intake (DMI), and blood hemoglobin [38,39]. High histidine levels in the diet may cause the conversion of this amino acid into glucose through the regulation of genes related to the gluconeogenic pathway in bovine hepatocytes [40]. One of the metabolic pathways related to different prenatal supplementation strategies is histidine metabolism. The highest histidine levels were found in the FP treatment (cows fed a protein energy supplement throughout the gestational period). These effects may imply a higher milk production and body weight of the dams (Table 3), directly affecting the performance of the offspring during the breastfeeding period.

The PC aa C42:2 and PC aa C26:0 were the phosphatidylcholines in dams and calves, respectively, selected for the discussion. However, more than half of the significant metabolites found for pre-delivery dams (PC aa C34:4, PC aa C38:3, PC aa C40:3, PC ae C38:1, PC ae C34:3, PC aa C40:4, PC aa C42:4, PC aa C42:2, PC ae C42:4, PC aa C42:6, and PC aa C36:2) and calves (PC aa C42:6, PC ae C38:4, PC aa C26:0, PC ae C40:4) between the treatments were from the phosphatidylcholines class. Phosphatidylcholines belong to a major class of lipids: the phospholipids (most abundant lipids in eukaryotic cells; [41,42]). These lipids are units of functional membranes, and their composition determines most properties of the cell membrane (fluidity, permeability, and thermal phase behavior; [43]). In humans, a correlation has been identified between pre-delivery maternal lipid metabolism and DNA methylation in the progeny. This may be responsible partly for health impacts and disease risks throughout the life of progenies [44]. The lipidome of cows and calves are similar to each other [45]. In our study, with the exception of PC aa C42:6 (in dams and calves), all other differentially expressed phosphatidylcholines showed higher levels in one of the treatments supplemented with protein and energy (PP or FP). The higher lipid levels found in these treatments can positively influence the fetus development during pregnancy and the reproductive parameters (ovarian follicle and corpus luteum function; [46]) in cows. The different levels of phosphatidylcholines found between prenatal treatments in calves may also affect their immune and inflammatory responses. This may occur because this class of lipids plays a role in the regulation of the inflammatory reactions, in addition to being related to cell membrane functions [47].

Carnosine is a dipeptide (β-alanyl-L-histidine) present in mammals. This metabolite is highly prevalent (about 99%) in skeletal muscle [48] and can also be found at low concentrations in the plasma of non-primate mammals [49]. When present in blood plasma, carnosine is transported into extra-intestinal tissues and cells, increasing its concentrations in skeletal muscle, brain, and heart [50]. The most important functions of carnosine are related to pH-buffering, activation of muscle ATPase to provide energy, antioxidant capacity, metal-ion (copper, zinc, and iron) chelation, and homeostasis [50,51]. In another study, correlations of carnosine with carcass quality and sensory scores in beef cattle were low, without significantly affecting the meat’s organoleptic properties [52]. However, more recently, an association of carnosine with low residual feed intake in Nellore animals was identified [53]. The greatest contrast in carnosine levels was found in the plasma of calves between the NP and FP treatments. This shows that progenies of cows that received protein-energy intake throughout pregnancy had lower carnosine levels compared to the control group. Thus, based only on carnosine levels, the feed efficiency of calves may be affected by prenatal nutrition [53].

Alanine is a non-essential amino acid that plays a role as raw material for glucose synthesis in the liver and muscles [54]. In cattle, alanine is one of the most abundant metabolites in the Longissimus thoracis and semimembranosus muscles [55] and the second most abundant metabolite in the liver [56]. The effects of alanine on productive traits in ruminants are still little discussed in the literature. However, it was demonstrated that, in an intrauterine environment with low nutrient and oxygen availability, sheep prioritized alanine and glutamine production for fetal organ growth and metabolism (a mechanism to sustain total energy needs; [57]). Here, we did not observe differences in alanine levels in dams, whereas the supplement of protein energy in the final third of pregnancy (PP) increased the alanine levels in calves compared to the treatment that received the same type of supplementation throughout the entire pregnancy (FP). These levels may be related to the fact that offspring in the PP treatment are more likely to present abnormalities in energy metabolism.

In another study, some biological processes (insulin secretion, PPAR signaling, and biosynthesis of amino acids) were identified due to the inclusion of vitamins and minerals in the diet as well as the rate of maternal weight gain of pregnant Angus cows [58]. In our study, histidine metabolism and beta-alanine metabolism were the significant biological processes found in both dams and calves. This may be indicative that epigenetic alterations caused by different prenatal supplementation approaches persist in the metabolism of the progenies throughout their life, affecting specific metabolic pathways. Studies on the epigenetic effects caused by maternal nutrition in cows and its impacts on metabolic pathways in the offspring should be carried out to elucidate some gaps still present in the literature.

## 4. Materials and Methods

### 4.1. Experimental Design

The study comprised 126 Nellore cows and their progenies. The dams were fixed-time artificially inseminated (FTAI) with semen from four sires and had their pregnancy diagnosis confirmed 30 days later.

The cows were blocked into three groups of 42 animals based on age, body weight (BW), and body condition score (BCS). The three groups were allocated in pasture paddocks of *Brachiaria brizantha* cv. *marandu*, equipped with a trough to supply feed supplements and water. The different prenatal nutrition strategies were: NP (control)—Not Programmed, PP—Partial Programming, and FP—Full Programming. All treatments received mineral supplementation (0.03% of BW), but only PP and FP received protein-energy supplementation equivalent to 0.3% of the average BW per day during pregnancy. The PP group was submitted to this nutritional protocol only in the final third of pregnancy, whereas dams in FP had supplementation upon pregnancy confirmation (30 days after FTAI) until calving (Table 4 and Table 5). Table 4 shows the supplements used, and Table 5 presents the nutrients of the pastures.

Forages were sampled by collecting five areas of 1 m^2^ in each paddock at random, avoiding areas with feces and invasive plants. The five samples were homogenized, and a single 300-g sample was obtained. Samples to determine dry matter (DM) were oven-dried by forced air ventilation at 65 °C for 72 h [59] and later ground in a 2-mm sieve for the bromatological and mineral analyses.

The bromatological and mineral analyses were conducted at the bromatology and mineral laboratory at the university. Crude protein [59], neutral detergent fiber (NDF; [60,61]), and the abundance of minerals were determined by Inductively Coupled Plasma Optical Emission Spectrometry (ICP-OES; [62]).

Table 6 shows the phenotypic effect of the different prenatal supplementation strategies on dams. After calving, protein-energy supplementation ceased, and all progenies (regardless of the prenatal nutritional treatment) were subjected to the same health protocols and nutritional managements, remaining together until weaning (210 ± 28 days). During this period (calving to weaning), the cows received the same mineral supplement (0.03% of BW) that they received during the pregnancy period and remained in an extensive pasture system (paddocks of *Brachiaria brizantha* cv. *Marandu*, as well as during the pregnancy period).

### 4.2. Plasma Sample Collection and Preparation

From the 126 cows, we selected 27 (9 per treatment randomly) and their respective offspring for plasma the metabolomics analysis. The cows were analyzed at early gestation (30 days of pregnancy; before receiving the nutritional treatments) and at pre-delivery (9 months of pregnancy; after receiving the nutritional treatments). Blood samples from calves were collected at an average of 30 ± 9.6 days of age. The blood was collected into EDTA-coated tubes (BD Vacutainer, São Paulo, SP, Brazil) from the jugular vein (conditioned on ice until the samples were processed in the laboratory). Blood samples were centrifuged for 10 min at 3000× *g* and 4 °C within 1 h after sampling. Plasma supernatants were transferred into fresh collection tubes, immediately snap frozen using dry ice, and stored at −80 °C until use.

### 4.3. Targeted Metabolomics

The metabolomics analysis was carried out by the Apex Science Company (Campinas, São Paulo, Brazil). Metabolites were quantified using AbsoluteIDQ^®^ p180 Kit (Biocrates Life Sciences AG, Innsbruck, Austria). The kit covers 188 metabolites, of which 21 are amino acids, 21 biogenic amines, 40 acylcarnitines (Cx:y), 14 lysophosphatidylcholines (lysoPC), 76 phosphatidylcholines (PC), and 15 sphingolipids (SMx:y). The placeholders x and y in these formulas represent the number of carbons and double bonds of all chains, respectively. The analysis uses two different mass spectrometric methods with isotope-labeled and other internal standards for the absolute quantification of metabolites. Mass detection and compound identification were performed by multiple reaction monitoring. The lysophosphatidylcholines, phosphatidylcholines, acylcarnitines, and the hexose were performed by flow injection analysis-tandem mass spectrometry (FIA-MS/MS). The amino acids and biogenic amines were derivatized using phenylisothiocyanate reagent (PITC; 5%) and analyzed by liquid chromatography-tandem mass spectrometry (quantified by stable isotopes; HPLC-MS/MS) in a positive mode using an AB SCIEX 4000 QTrap mass spectrometer (AB SCIEX, Darmstadt, Germany) with electrospray ionization. More specifically, the analyses of amino acids and biogenic amines were based on PITC derivatisation, separation of metabolites on a Waters Acquity UHPLC BEH18 C18 reversed-phase column (Waters, Vienna, Austria) using water and acetonitrile with 0.1% formic acid as mobile phases, and quantification on a Triple-Stage Quadrupole tandem mass spectrometer with electrospray ionization in the presence of internal standards. The analyses were performed in a 96-well-plate format, allowing for measurements in batches of 81 samples at one time.

Data analysis for metabolite quantification and quality assessment was performed using MetIDQ^®^ software Version 1.0, which is part of the AbsoluteIDQ^®^ p180 kit (Biocrates Life Sciences AG, Innsbruck, Austria). Metabolite concentrations were calculated using internal standards. The concentration of each metabolite was measured in μM. The metabolite-specific limits of detection (LOD), lower limits of quantification, and upper limits of quantification of the assay were experimentally determined by Biocrates (Table S4).

### 4.4. Statistical Analysis

Data processing and the univariate analysis (analysis of variance; ANOVA) were performed using the R software environment (version 4.1.2). Metabolites with more than 70% of samples below LOD were removed from the dataset (Early gestation (dams) = 129 metabolites remaining; Pre-delivery (dams) = 128 metabolites remaining; Calves = 124 metabolites remaining). The LOD values that remained in the metabolome after filtering were replaced by the mean of each variable. Two models were implemented through the “LM” function in R.

The statistical model used in the metabolomics analysis of dams:

(1) $$Y_{\mathrm{jk}}=\;\mu +{\;\beta }_{1}{\mathrm{Age}}_{d1}+{\;\mathrm{Treat}}_{j}+e_{\mathrm{jk}}$$

The statistical model used in the metabolomics analysis of offspring:

(2) $$Y_{\mathrm{ijk}}=\;\mu +{\;\beta }_{1}{\mathrm{Age}}_{c1}+{\;\mathrm{Treat}}_{j}+Sex_{i}+e_{\mathrm{ijk}}$$

where: Y_ijk_ and Y_jk_ are the observed metabolite from kth animal, recorded on jth treatment of sex ith (only in the model for offspring); μ is only a constant; β_1_ is the regression coefficient of covariate for animal age; Age_d1_ is the observed value for the age of dams of kth animal; Age_c1_ is the observed value for the age of calves of kth animal; Treat_j_ is the fixed effect of jth treatment; and e_ijk_ is the residual random term. The residuals were tested for normality (Shapiro–Wilk test) and for homoscedasticity (Levene’s test) and the differences between treatments were considered significant when *p* value was ≤0.05 by the Tukey–Kramer test. Data that did not meet these prerequisites were transformed into log10, square root, or cubic root, depending on which transformation met the requirements (homoscedasticity and normality of the residuals). The evaluation of initial metabolome of dams was performed to investigate if the initial metabolomics profiles among treatments were at the same levels, since initial phenotypic traits were similar (Table 6).

In addition, the metabolite concentration table was uploaded to MetaboAnalyst 5.0 [63] and the data were Auto-scaled (mean-centered and divided by the standard deviation of each variable) before the analysis. The supervised method (Partial least squares discriminant analysis (PLS-DA)) and the unsupervised method (Principal component analysis (PCA)) were performed. The PLS-DA, PCA, and enrichment analyses were performed only for dams in the pre-delivery stage and for calves since the aim of these analyses was to find variables and processes related to a group, treatment, or phenotype. However, we carried out a PCA for time to evaluate the clustering between dams without the treatment (early gestation) and after receiving the treatments (pre-delivery). Cross-validation was performed for PLS-DA using the leave-one-out cross-validation method (LOOCV) and the performance measure “accuracy” (dams: R^2^ = 0.98, accuracy = 0.70; calves: R^2^ = 0.88, accuracy = 0.41). The variable importance in the projection (VIP) plot was used to rank the metabolites based on their importance in the treatments. Metabolites with the highest VIP values were the most powerful group discriminators. The VIP values > 1 were significant, while the VIP values > 2 were highly significant. The enrichment analysis was performed to identify the most relevant biological processes associated with differentially expressed metabolites (identified in univariate analysis) using MetaboAnalyst 5.0 (based on the Kyoto Encyclopedia of Genes and Genomes database). Biological processes with *p* value < 0.1 were considered significant.

## 5. Conclusions

Fetal programming altered the metabolome of dams and their offspring, especially in terms of protein metabolism. Significant metabolites affect productive traits of interest in livestock as well as important biological pathways. Two of these pathways (histidine metabolism and beta-alanine metabolism) are common to both dams and progenies in Nellore cattle, which may be indicative of transgenerationally transmitted epigenetic alterations.

## Figures and Tables

**Table 1:** Mean concentration of significant metabolites ± standard error of the mean for cows in early-gestation (initial period, before receiving the treatments) with their respective *p* values.

Metabolites (µM)	NP	PP	FP	*p* Value
PC aa C30:0	1.44 ± 0.05 ^a,b^	1.26 ± 0.04 ^a^	1.58 ± 0.06 ^b^	<0.001
Histidine	61.3 ± 4.03 ^a^	43.1 ± 3.31 ^b^	57.5 ± 2.83 ^a^	0.003
PC ae C30:2	254.1 ± 16.0 ^a,b^	235.2 ± 8.11 ^a^	289.3 ± 6.17 ^b^	0.004
PC ae C30:0	110.3 ± 5.80 ^a,b^	101.0 ± 3.76 ^a^	127.0 ± 7.51 ^b^	0.009
PC ae C30:1	413.2 ± 37.3 ^a^	419.1 ± 21.4 ^a,b^	505.1 ± 19.9 ^b^	0.044

The small letters overwritten represent the significant contrasts. NP—not programmed; PP—partial programming; FP—full programming.

**Table 2:** Mean concentration of significant metabolites ± standard error of the mean for cows in pre-delivery period (after receiving the prenatal nutritional treatments) with their respective *p* values.

Metabolites (µM)	NP	PP	FP	*p* Value
SDMA	0.86 ± 0.03 ^a^	0.65 ± 0.03 ^b^	0.67 ± 0.04 ^b^	0.001
PC aa C34:4	10.5 ± 1.21 ^a^	17.9 ± 2.09 ^b^	17.0 ± 1.82 ^b^	0.002
PC aa C38:3	61.9 ± 4.35 ^a^	89.6 ± 6.73 ^b^	97.8 ± 10.6 ^b^	0.003
PC aa C40:3	4.14 ± 0.38 ^a^	6.71 ± 0.59 ^b^	7.35 ± 0.84 ^b^	0.005
Taurine	16.2 ± 2.03 ^a^	30.7 ± 4.54 ^b^	23.0 ± 1.86 ^a,b^	0.005
Glutamic acid	48.0 ± 3.34 ^a^	55.3 ± 2.66 ^a,b^	70.2 ± 6.06 ^b^	0.005
PC ae C38:1	12.8 ± 1.00 ^a^	14.4 ± 2.02 ^b^	14.4 ± 2.31 ^b^	0.009
PC ae C34:3	30.6 ± 3.60 ^a^	51.7 ± 5.92 ^b^	44.1 ± 4.29 ^a,b^	0.014
PC aa C40:4	11.9 ± 1.01 ^a^	14.8 ± 1.09 ^a,b^	17.0 ± 1.32 ^b^	0.015
PC aa C42:4	60.7 ± 5.83 ^a^	79.3 ± 8.37 ^a,b^	92.0 ± 8.31 ^b^	0.030
PC aa C42:2	51.0 ± 5.67 ^a^	69.0 ± 5.75 ^b^	55.1 ± 4.22 ^a,b^	0.035
PC ae C42:4	0.55 ± 0.05 ^a^	0.75 ± 0.06 ^b^	0.73 ± 0.06 ^a,b^	0.037
PC aa C42:6	0.92 ± 0.06 ^a^	0.81 ± 0.06 ^a,b^	0.70 ± 0.05 ^b^	0.040
Histidine	74.2 ± 4.17 ^a,b^	70.3 ± 4.91 ^a^	88.2 ± 5.36 ^b^	0.042
Carnosine	25.4 ± 2.03 ^a^	31.1 ± 2.39 ^a,b^	33.7 ± 2.80 ^b^	0.044
PC aa C36:2	289.1 ± 41.1 ^a^	457.2 ± 54.3 ^b^	414.0 ± 48.31 ^a,b^	0.048

The small letters overwritten represent the significant contrasts. NP—not programmed; PP—partial programming; FP—full programming.

**Table 3:** Mean concentration of significant metabolites ± standard error of the mean for offspring at 30 days of age submitted to different prenatal nutrition approaches (NP, PP and FP) with their respective *p* values.

Metabolites (µM)	NP	PP	FP	*p* Value
PC aa C42:6	1.45 ± 0.15 ^a^	1.24 ± 0.07 ^a,b^	0.96 ± 0.06 ^b^	0.010
PC ae C38:4	6.14 ± 0.42 ^a^	8.43 ± 0.59 ^b^	7.50 ± 0.51 ^a,b^	0.011
Carnosine	31.8 ± 3.20 ^a^	30.3 ± 3.46 ^a,b^	20.6 ± 1.88 ^b^	0.030
Alanine	261.0 ± 17.8 ^a,b^	279.2 ± 14.8 ^a^	226.0 ± 8.40 ^b^	0.048
PC aa C26:0	7.64 ± 0.24 ^a,b^	9.07 ± 0.85 ^a^	6.57 ± 0.72 ^b^	0.049
PC ae C40:4	1.56 ± 0.12 ^a^	2.09 ± 0.16 ^b^	1.94 ± 0.21 ^a,b^	0.049

The small letters overwritten represent the significant contrasts. NP—not programmed; PP—partial programming; FP—full programming.

**Table 4:** Ingredients and nutrients content of the supplement for dams.

Ingredients/Nutrients	Mineral Supplement	Protein-Energy Supplement
Corn (%)	35.00	60.00
Soybean meal (%)	-	30.00
Dicalcium phosphate (%)	10.00	-
Urea 45% (%)	-	2.50
Salt (%)	30.00	5.00
Minerthal 160 MD (%) *	25.00	2.50
Total digestible nutrients (%)	26.76	67.55
Crude protein (%)	2.79	24.78
Non-protein nitrogen (%)	-	7.03
Acid detergent fiber (%)	1.25	4.76
Neutral detergent fiber (%)	4.29	11.24
Fat (%)	1.26	2.61
Calcium (g/kg)	74.11	6.20
Phosphorus (g/kg)	59.38	7.24

* Mineral premix composition (Minerthal company, Sao Paulo, Brazil): Calcium = 8.6 g/kg; Cobalt = 6.4 mg/kg; Copper = 108 mg/kg; Sulfur = 2.4 g/kg; Fluorine = 64 mg/kg; Phosphorus = 6.4 g/kg; Iodine = 5.4 mg/kg; Manganese = 108 mg/kg; Selenium = 3.2 mg/kg; Zinc = 324 mg/kg; Sodium monensin = 160 mg/kg [8].

**Table 5:** Nutrients in pastures consumed by dams in the different groups (mean ± standard error of the mean).

Forage Nutrients	NP	PP	FP
CP % (crude protein)	7.38 ± 0.70	7.82 ± 0.93	7.40 ± 0.93
TDN % (total digestible nutrients)	63.1 ± 0.59	64.1 ± 0.95	61.4 ± 0.86
NDF % (neutral detergent fiber)	59.0 ± 1.49	61.4 ± 2.06	58.4 ± 1.67
Ca % (calcium)	0.38 ± 0.04	0.35 ± 0.02	0.39 ± 0.03
P % (phosphorus)	0.19 ± 0.01	0.19 ± 0.01	0.17 ± 0.01

NP—not programmed; PP—partial programming; FP—full programming.

**Table 6:** Average of BW (kg), subcutaneous fat thickness (SFT; mm) and body condition score (BCS) ± standard error of the mean of Nellore cows submitted to different maternal nutrition approaches (NP, PP and FP) with their respective *p* values.

Traits	NP	PP	FP	*p* Value
Initial BW (kg)	461.1 ± 6.90	451.2 ± 9.38	454.1 ± 8.76	0.85
Pre-delivery BW (kg)	508.0 ± 7.23 ^a^	524.2 ± 9.07 ^a^	541.2 ± 10.1 ^b^	<0.01
Initial SFT (mm)	4.28 ± 0.61	4.31 ± 0.61	4.33 ± 0.61	0.92
Pre-delivery SFT (mm)	7.23 ± 0.66 ^a^	9.24 ± 0.67 ^a^	12.5 ± 0.98 ^b^	<0.01
Initial BCS	4.50 ± 0.09	4.60 ± 0.12	4.50 ± 0.09	0.34
Pre-delivery BCS	5.40 ± 0.13 ^a^	5.60 ± 0.13 ^a,b^	5.90 ± 0.13 ^b^	0.04

The small letters superscripted represent the significant contrasts. NP—not programmed; PP—partial programming; FP—full programming.

## Data Availability

The datasets generated during and/or analyzed during the current study are available from the corresponding author on reasonable request due to restrictions on privacy.

## Supplementary Materials

The following supporting information can be downloaded at: https://www.mdpi.com/article/10.3390/metabo12040347/s1, Table S1: Mean concentration (µM) of significant and not significant metabolites ± standard error of the mean of cows in early-gestation (initial period, before receiving the treatments) with their respective *p* values; Table S2: Mean concentration (µM) of significant and not significant metabolites ± standard error of the mean of cows in pre-delivery period (after receiving the prenatal nutritional treatments) with their respective *p* values; Table S3: Mean concentration (µM) of significant and not significant metabolites ± standard error of the mean of offspring at 30 days of age submitted to different prenatal nutrition approaches (NP, PP and FP) with their respective *p* values; Table S4: Limits of quantification and detection of metabolites of the AbsoluteIDQ p180 kit.
